# Verification and Evaluation of a Visual Reaction System for Badminton Training

**DOI:** 10.3390/s20236808

**Published:** 2020-11-28

**Authors:** Kuei-Pin Kuo, Hsun-Heng Tsai, Ching-Yi Lin, Wei-Te Wu

**Affiliations:** 1 Office of Physical Education, National Ping-Tung University of Science and Technology, Neipu 912, Pingtung County, Taiwan; kweibin@mail.npust.edu.tw (K.-P.K.); lincy622@gmail.com (C.-Y.L.); 2 Department of Biomechatronics Engineering, National Ping-Tung University of Science and Technology, Neipu 912, Pingtung County, Taiwan

**Keywords:** agility, competitive environment, footwork drills, simulation

## Abstract

The two aims of this study were (1) designing and developing an affordable visual reaction system for badminton training that monitors and provides instant feedback on agility; and (2) to measure and improve the footwork and movement of badminton players and output useful reference data. Ten junior high school badminton players were invited to serve as the subjects of this study. They participated in a three-week (nine sessions) training program. Training was primarily in the form of fixed or random footwork drills. Timed tests were performed before and after each session to measure the players’ agility in performing six-point and four-point footwork drills. The results were compared to the training effects calculated using dependent-sample *t*-tests. In addition, the long-term durability and functionality of the training system were tested. The training system was able to maintain stable and reliable training and evaluation operations for extended periods. Results showed significant improvements in the visual reaction time (*p* = 0.003) and agility (*p* = 0.001) of players. The proposed training system is an affordable option for training and monitoring, evaluating, and recording training performance. It can accurately record movement and response times and simulate competitive environments.

## 1. Introduction

As of 1 January 2006, badminton games are played to 21 using a rally-point system rather than the previous serve-point system. The new system of scoring whenever the shuttlecock touched the ground accelerated the pace of the game and placed greater emphasis on the all-round skill of the players. Therefore, players were forced to change and adjust their strategies that were geared towards the old rules to speed-based strategies that focused on aggressive yet stable short-serve offensive, serve-receive offensive, and fast offensive and defensive tactics [1,2]. Observation of current world badminton tournaments shows that players’ competitive training regimens focus on “all-round skill, strength development, aggressive offensive, balanced offense and defense, and quick victory” with “proactiveness, speed, and accuracy” being the main tactics (cited from [3]). Athletes who demonstrate the ability to move and change direction quickly and perform continuous activations, terminations, and jumps are more able to maximize technical performance and score points [4]. Playing fast-paced offense strategies and creating net shots are the trends and strategies of today’s badminton games [5]. Therefore, badminton players play short, fast-paced games with high-intensity intervals and are required to execute strategies and launch smashes while rallying with the opponent, followed by a brief rest period between games. Chang and Lu [6] analyzed individual rallies (from serve to score) and games (from 0 to 21) and found that a match lasts between 18 and 90 min, with an average match time of 33 min, and that players average seven shots per rally. Tsai et al. [7] cited Manrique and Gonzalez [8], asserting that a rally in badminton lasts between 4.57 and 8.86 s, wherein players’ lactic acid concentrations reach 2.4 to 5.1 mmol/L and heart rates reach 186 to 201 beats/min. These measurements are equivalent to the maximum heart rate of in-game athletes. Phomsoupha and Laffaye [9] found that in the current time structure of badminton matches, a woman’s single match is roughly 23 min. Each rally is approximately 6.1 s, followed by a brief preparation and rest period of 14 s. The shot rate in badminton is roughly 0.89 s. The conclusions of the aforementioned studies show that athletes perform a series of high-intensity fast-paced strokes and movements during a game, which elevates average cardiovascular loads. In a short reaction window, athletes must focus, analyze the situation, predict their opponent’s movement, and combine reactions and tactics to maximize performance. These operations constitute a badminton game [9]. Therefore, badminton players must be able to move freely and pace themselves during a game to gain the upper hand and create opportunities to steadily and fluidly launch smashes [10]. To maintain the upper hand and create scoring opportunities, badminton players must have excellent footwork. They must also demonstrate the visual acuity and reflexes to determine stroke direction and timing, thereby enhancing their competitiveness. In short, the fundamental development of badminton players must focus on fostering excellent visual reaction and agility. With these prerequisites, the subsequent development of tactics and strategies would be more effective.

Lu and Chang [10] asserted that badminton footwork comprises five basic components, namely, shuffle, hitch, stride, drive, and hop. The coordinated application of these components helps players gain the upper hand on the court. Footwork drills in specialized badminton training programs include full-court six-point footwork drills, forward/backward runs, and shuttle runs. Players familiarize themselves with all possible changes in trajectory and apply footwork strategies accordingly. All strategies are based on six-point footwork [11]. At the same time, players must remain focused and continuously track the movement of their opponent and the trajectory of the shuttlecock [12]. Players are engaged continuously in four processes when rallying with their opponent. These processes are activation, breaking, changing direction, and acceleration. This ability is known as the agility required to maintain excellent footwork in badminton [13]. Therefore, superior visual reaction and footwork are crucial to maximizing agility, allowing players to move quickly on the court and make sufficient returns [14].

Agility is an essential ability in the majority of sports and often the key to victory. Evading attacks in Taekwondo and boxing, dribbling past opponents in soccer, shifting horizontally and making turn shots in billiard, and performing six-point footwork in badminton require excellent agility to be performed effectively [15,16]. Badminton footwork is a regularity and key to improving technique. The core element in badminton footwork is activation. A fast activation helps players move into position quicker. In badminton, speed is determined by reaction of players to starting a movement. Therefore, having a high level of agility is essential for badminton players. Many badminton-related studies on footwork training and reaction ability have been published. For example, [1,13,15,17,18] all found that reaction time, return position, and footwork position and connection significantly influenced badminton performance. However, footwork and reaction training has always been centered on repetitive self-training exercises. In large teams, one or two coaches are unable to monitor the training outcomes of all the players. Players are left to self-train and self-monitor their performance. Coaches are unable to adequately assess players’ efforts. In this context, the cross-disciplinary system for training badminton footwork and reaction designed and developed in this study uses visual cues and movement reactions to help badminton players perform footwork drills, engage in physical training, and foster tactics. It can also be combined with supervised striking exercises, thereby maximizing training effectiveness through the integration of smart functions and sports science. In today’s highly competitive tournament conditions, grounded basic training is key to enhancing competitiveness. A technology-assisted monitoring and analysis system to share the workload of coaches and provide accurate player training data is hugely beneficial for improving player performance. The proposed training system was widely accepted by badminton coaches. In addition to training and evaluation, the proposed system can also be used for screening talent. It can even be applied to other sports.

In this study, we evaluated the feasibility of self-designing and self-developing a visual reaction system for badminton training to assist in fundamental badminton training and improve players’ agility, including cognitive (visual scanning), physical (strength/power/reactive strength), and technical three factors [19,20]. The two main objectives of this study were (1) to adopt a cross-disciplinary approach in designing and developing an affordable visual reaction system for badminton training that monitors and provides instant feedback on agility and basic six-point footwork training conditions; and (2) to measure and improve the footwork and movement of badminton players and output useful reference data.

This study hypothesized that the visual reaction system has been tested by intra-class correlation (ICC > 0.7), and low coefficient of variation (CV) to verify the reliability and validity as an experimental tool. After 3 weeks of training, the badminton players have made significant progress on reaction time, movement time, and the T-shaped run.

## 2. Methodology

### 2.1. Participants

**Figure 1:**
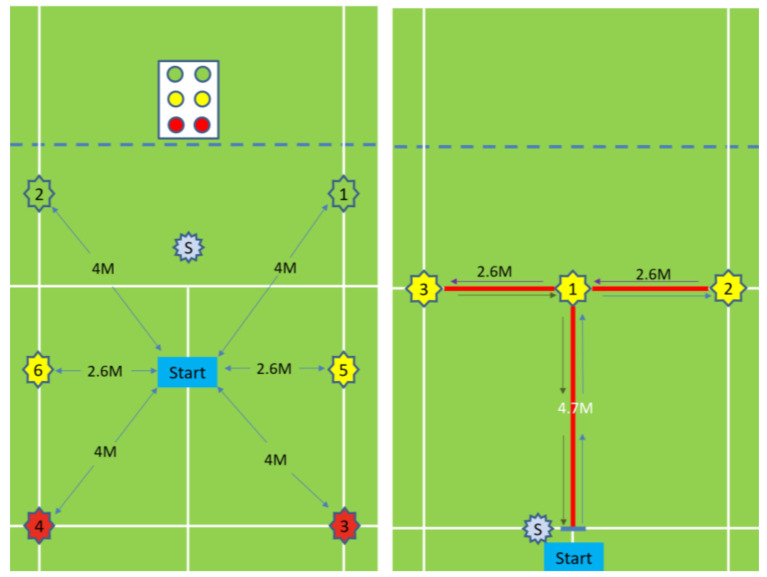
Six-point and T-shaped footwork drills and measurement method.

Ten junior high school badminton school team players (nine men and one woman) were invited to participate in the experiment. The average height of participants was 162.2 ± 4.9 cm, and their average weight was 52.7 ± 7.4 kg. None of the players had sustained lower limb injuries in the past six months. The participants engaged in three training sessions a week for three weeks for a total of nine sessions. The length of each session was between 8 and 12 min. The training content was primarily fixed and random six-point footwork drills (Figure 1). Players underwent two randomized six-point runs (84.8 m) and two t-shaped runs (39.6 m) before and after each session to measure their performance and collect their visual reaction and overall movement times.

### 2.2. Tools and Procedures

#### 2.2.1. Visual Reaction System for Badminton Training

**Figure 2:**
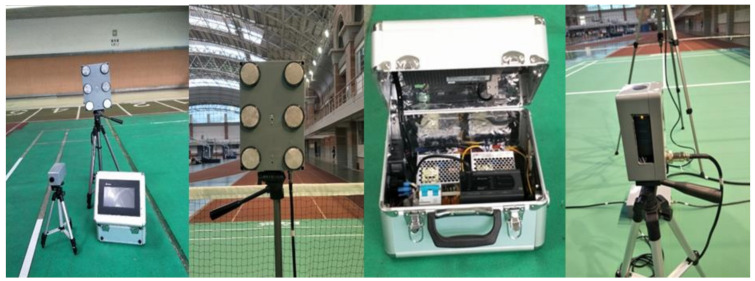
The badminton visual reaction system for badminton training and components.

The primary function of the proposed visual reaction system for badminton training (Figure 2) was to administer and monitor specialized badminton footwork drills. The system provided a series of visual cues to prompt agile reactions and rapid movements. LED lights, a programmable controller, and optical sensors were used for training and measurements. The information/training system provided visual cues to the participants by lighting locations on the court. The players responded to these cues by moving to the marked locations. The system then determined and recorded various time parameters during the session. These measurements serve as post-training feedback, allowing coaches to modify their training programs.

**Figure 3:**
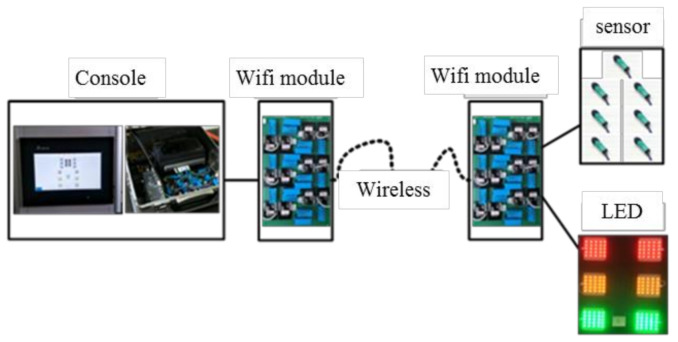
Architectural diagram of the wireless transmission system.

**Figure 4:**
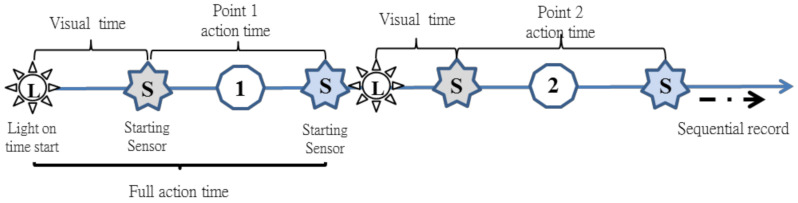
Measurement procedure for the six-point footwork drills and tournament training program.

**Figure 5:**
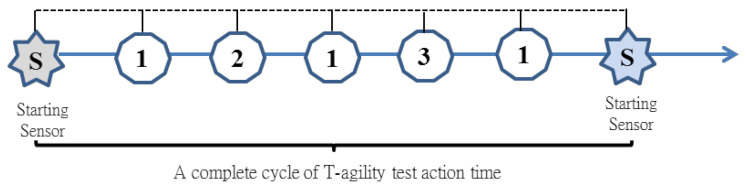
Agility measurement procedure for self-administered T-shape footwork drills.

The training system comprised an industrial-grade programmable logic controller (PLC, Delta, Taiwan) and a human-machine interface (HMI, Delta, Taiwan). An architectural diagram is illustrated in Figure 2. The PLC and HMI constituted the primary control platform. The RS232 communication protocol was adopted for the transmission of data and HMI information. Internal programming was written using the sequential function chart (SFC). A multi-core control cable configuration was adopted for the transmission signals of the infrared sensors and LEDs. The system has the function of wireless transmission in an obstacle-free area of 100 m. Signals were transmitted over a universal radiofrequency between 2400 and 2480 MHz. Sensor and LED signals from various points were transmitted wirelessly to the control platform through the module (Figure 3). The proposed training system primarily featured training and measurement functions. The training functions included fixed six-point footwork training, random six-point footwork training, and tournament simulation training. The system measured visual reaction time, directional movement time, overall movement time, tournament movement time, and T-shaped agility (Figure 4 and Figure 5) and provided a spreadsheet and graphs of the training results.

#### 2.2.2. Reliability and Validity Analysis

Before the ten junior high school badminton school team players formally conduct the test, sixty college students conducted two random six-point running trainers (84.8 m) and two T-shaped running trainers (39.4 m). To analyze the reliability and validity of the visual reaction system as a detection tool. The tests were completed in stages with sufficient rest between each item. The training system was used to record the participants’ performance, and a manual stopwatch was used to record the completion times. None of the players had sustained lower limb injuries in the past six months. Referencing [21], the reliability of the data was measured using SPSS 20.0 and ICC tests. In addition, the mean and standard deviation values of each completion time were calculated to determine the CV.

#### 2.2.3. Training Content

We designed fixed and random six-point (Figure 1) footwork drills and simulated tournament footwork drills administered by the proposed training system to determine whether the proposed training system is capable of supporting the training of badminton coaches. Training was conducted in a way that reflected real-world conditions. Each session was between 8 and 12 min (excluding rest time). The details of the training program were tabulated in Table 1.

#### 2.2.4. Data Collection and Statistical Analysis

In this study, the proposed visual reaction system for badminton training was adopted as the research tool to collect the reaction data of players performing pre-training and post-training six-point footwork and T-shaped footwork drills. The training system recorded all visual reaction times, overall movement times, and agility (T-shaped movement times). The measurements served as a reference for evaluating training performance. The SPSS 20.0 software was employed for statistical analysis. Dependent-sample *t*-tests were performed to evaluate training performance. The level of statistical significance was set at α = 0.05.

## 3. Results and Discussion

### 3.1. Mode Analysis of the Visual Reaction System for Badminton Training

**Figure 6:**
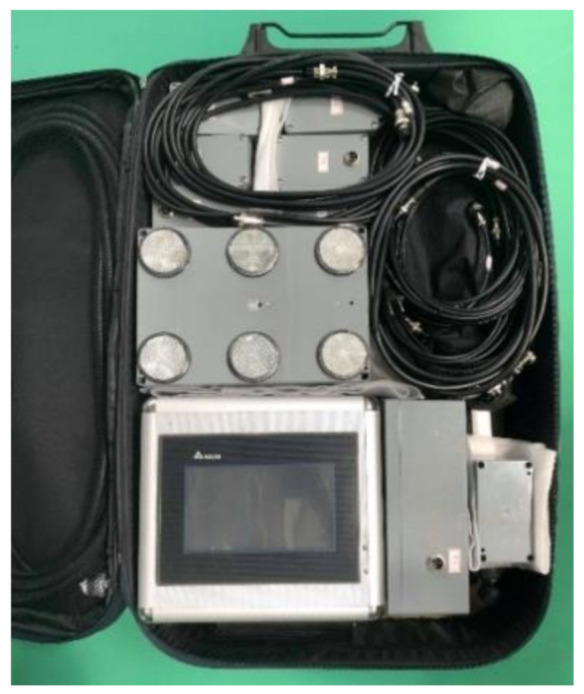
The packed system.

The visual reaction system for badminton training was improved from the 1.0 wired system to the 2.0 wireless system. The focus of the design was affordability, durability, and weight (Figure 6). Following improvement and testing, the dimensions of the wireless system were reduced by roughly 30% compared to its predecessor and fit into a 24-inch travel case, allowing for easy transport and assembly. In terms of practicality, improvements also focused on the core structure. An industrial-grade PLC was selected for the core for its humidity and temperature resistant properties and long-operation capabilities, thereby reducing downtime and the likeliness of failure. We performed a stability test by continuously monitoring 60 participants while they performed six-point and T-shaped footwork drills. The system was continuously operated for six hours without crashing or malfunction. Therefore, the system met durability and stability expectations. In terms of the peripheral sensing components and wireless module, basic optical sensors were used to reduce cost. These components can be replaced quickly if they malfunction or are damaged.

In terms of the different training modes, a fixed six-point footwork training mode, a randomized six-point footwork training mode, T-shaped agility training mode, and tournament training mode have been completed. Five Group A players were invited to test the tournament mode. Each drill comprised 30 rallies, with eight beats per rally and a rest period of 10 s between rallies. Test results showed that the system operated stably and continuously without malfunctioning. The measurements, records, analysis, and feedback of the reaction times, movement times, and overall movement times met the expectations of the research team. The players expressed that a systematic training program is beneficial for beginner and intermediate footwork training, reaction training, and endurance training. The system also effectively helps coaches in designing a training program and provides them with evaluatory data on their players.

In terms of reliability and validity, completion times were simultaneously measured with a training device and a stopwatch. The average readings and standard deviation values are tabulated in Table 2. An analysis of the coefficient of variation revealed that the training device produced less variation than the stopwatch for both the randomized six-point footwork drills and T-shaped footwork drills. Interclass correlation coefficient test results indicated similar findings. Results of the reliability analysis indicated that both the visual reaction system for badminton training and the stopwatch had excellent ICCs for both tests (ICC = 0.95 and 0.96). Typically, ICCs should be within a range of 0 and 1. An ICC value smaller than 0.4 denotes poor repeatability, while an ICC value higher than 0.75 denotes excellent repeatability. Therefore, the results validated that the proposed training system had excellent repeatability. Furthermore, the CV values of the proposed system were lower than those of the stopwatch for both the randomized six-point footwork drill and T-shaped footwork drill, suggesting that the proposed system was more stable and accurate than the stopwatch. ICC and CV analysis results indicated that the proposed system was a reliable detection tool. The results of this study were similar to those proposed by Chang et al. [18].

### 3.2. Training Effectiveness

**Figure 7:**
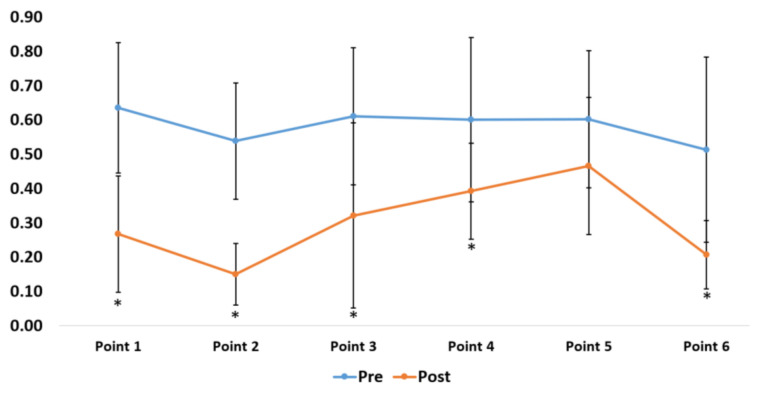
A comparison of the visual reaction times at various points in the six-point footwork drill.

**Figure 8:**
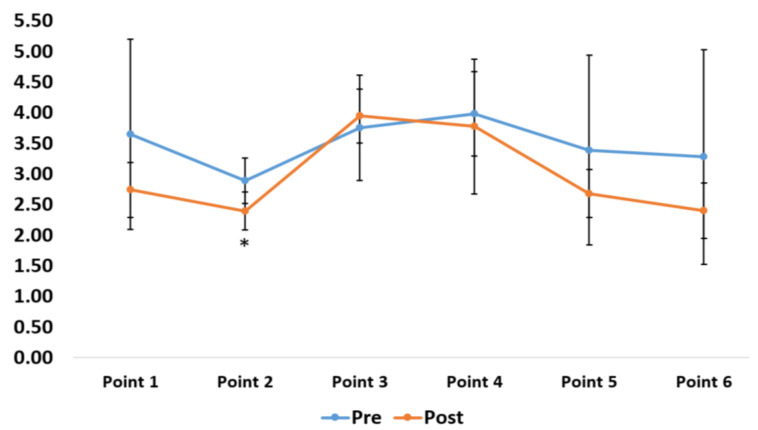
A comparison of the training at various points in the six-point footwork drill.

The junior high school badminton players underwent nine training sessions over three weeks. The average reaction time in the pre-test was 0.583 ± 0.042 s, and the average reaction in the post-test was 0.301 ± 0.107 s. The average time for overall six-point footwork in the pre-test was 20.94 ± 3.98 s, and the average time in the post-test was 17.93 ± 2.09 s. The average time for agility in performing T-shaped footwork in the pre-test was 8.94 ± 1.07 s, and the average time in the post-test was 7.74 ± 0.56 s. Dependent-sample *t*-tests were performed to validate training effectiveness (visual reaction time, overall movement time, and agility; Table 3). Test results showed significant improvements in visual reaction time (*t* = 4.09) and agility (*t* = 5.13). Only overall movement time failed to achieve significant statistical differences. Although the pre-test mean values and post-test mean values failed to achieve significant differences, there was a slight improvement in the times. Significant improvement could be achieved with a longer training period. The training results for the six-point footwork drills showed that the times for point five, which represented center-to-right movement, failed to achieve significant improvement. The other points showed significant improvement (Figure 7). In terms of overall movement time, only the movement time of point two, which represented center-to-forward left movement, achieved significant improvement. No significant improvements were observed in the other points (Figure 8).

The findings of this study show that the visual reaction, footwork, and agility performance of junior high school badminton players significantly improved after three weeks of undergoing footwork drills administered by the visual reaction system for badminton training. A number of previous studies indicated that agility training improves the ability of the neuromuscular system to control movements and neural adaptability, thereby enhancing motor skills and responsiveness [22,23,24]. The purpose of this study was to test the effectiveness of a visual reaction system for badminton training designed and developed by the research team in administering and monitoring badminton footwork drills. The outcomes of a three-week footwork training intervention were similar to those proposed by Chang et al. [25] and Kao et al. [26]. Therefore, we validated that the proposed training system can serve as an auxiliary tool for footwork training intervention and can effectively improve reaction time, movement time, and agility.

This study aimed to develop a visual reaction system to administer badminton footwork drills and facilitate player training. The system achieves smart detection and computation by issuing visual cues and detecting movement using optical sensors. This function helps coaches administer badminton footwork and movement drills and record player performance in real-time. The various movement parameters are provided to coaches and players to help them with their badminton training. The proposed system is an innovative smart training product. Similar products are available overseas. However, the hefty price tags of these systems are generally beyond the financial capability of grass-roots training stations and schools in Taiwan. Therefore, they are forced to continue applying traditional training methods that are resource-intensive and difficult to monitor scientifically. The proposed system was designed and developed using proprietary technologies and market-available components, allowing the research team to maintain production costs below NT $100,000. The system provides an affordable means for grass-roots training stations or schools to enhance training performance. After testing, the proposed system was able to stably administer training workloads without malfunction while providing training feedback in real-time for coaches and players to review and revise. At present, all research and development objectives have been fulfilled. We are currently moving towards product commercialization and system refinement, such as adding databases for smart corrections and providing training plans, thereby allowing junior high school badminton training to be more scientific, improving the entry-level training performance, and creating an internationally competitive product.

## 4. Conclusions

A statistical analysis of the test times recorded by the proposed training system, the visual reaction system for badminton training, achieved excellent ICCs and low CVs, suggesting that the system is a reliable measurement tool. In terms of training performance, a training program comprising nine sessions over three weeks effectively improved the visual reaction time and agility of junior high school badminton players. Although no significant improvements in overall movement time were observed, a level of improvement was achieved. We believe that significant improvements can be achieved by extending the training period.

The proposed visual reaction system for badminton training has yet to be perfected. Nonetheless, it can already apply sports science to training. The research team will continue to revise and refine the system to develop a system with artificial intelligence (AI) and machine-learning capabilities, allowing the system to determine shortcomings as players train, devise improvement strategies, and allow training to be more scientific and intelligent.

## Figures and Tables

**Table 1:** Training program and content.

Distribution Item	Week	Week 1	Week 2	Week 3
Fixed direction	1	1C*3n*2N (r = 7”, R = 90”)	2C*2n*2N (r = 8”, R = 120”)	2C*2n*2N (r = 10”, R = 120”)
	3	2C*2n*1N (r = 7”, R = 120”)	2C*2n*2N (r = 10”, R = 120”)	2C*2n*3N (r = 10”, R = 120”)
Random direction	1	2C*1n*2N (r = 10”, R = 120”)	1C*4n*3N (r = 10”, R = 120”)	3C*2n*2N (r = 15”, R = 150”)
	3	1C*4n*2N (r = 7”, R = 120”)	3C*2n*1N (r = 15”, R = 120”)	2C*3n*3N (r = 15”, R = 150”)
Tournament mode	5	25 rallies 1 rally 4 points 8 s rest	30 rallies 1 rally 6 points 10 s rest	35 rallies 1 rally 8 points 10 s rest

Note: C = complete 6 points; HRmax = maximum heart rate; n = intragroup count; N = intergroup count; r = intragroup rest; and R = intergroup rest.

**Table 2:** Reliability and validity analysis of the measurement produced by the training system and a stopwatch.

Drill	Group	Mean (s)	Standard Deviation (±)	CV	ICC
Randomized six-point footwork drill	Stopwatch	43.89	4.42	19.49	0.95
	Training device	43.48	4.21	17.76	
T-shaped footwork drill	Stopwatch	16.40	2.05	4.21	0.96
	Training device	15.59	1.96	3.84	

**Table 3:** A comparison of the various pre-training and post-training indicators.

Item	Stage	Mean	(±) SD	*t*-Value	*p*-Value
Visual reaction time	Pre	0.583	0.042	4.09	0.003 *
	Post	0.301	0.107		
Overall movement time	Pre	20.94	3.98	1.71	0.126
	Post	17.93	2.09		
Agility	Pre	8.94	1.07	5.13	0.001 *
	Post	7.74	0.56		

* *p* < 0.05.
